# Insights into the history of olingos, *Bassaricyon* (Carnivora, Procyonidae) from the Guiana Shield

**DOI:** 10.3897/zookeys.1286.186604

**Published:** 2026-07-24

**Authors:** Cibele R. Bonvicino, Maria Julia Cardoso, Filipe Souza-Gudinho

**Affiliations:** 1 Laboratório de Biologia e Parasitologia de Mamíferos Silvestres Reservatórios, Instituto Oswaldo Cruz, Fiocruz, Av. Brasil, 4365, Rio de Janeiro, RJ, Brazil Postgraduate in Genetics, Universidade Federal do Rio de Janeiro Rio de Janeiro Brazil; 2 Postgraduate in Genetics, Universidade Federal do Rio de Janeiro, Avenida Carlos Chagas Filho, 373, Bloco A, Rio de Janeiro, RJ, Brazil Laboratório de Biologia e Parasitologia de Mamíferos Silvestres Reservatórios, Instituto Oswaldo Cruz Rio de Janeiro Brazil; 3 Setor de Mastozoologia, Museu Nacional/UFRJ, Quinta da Boa Vista, Rio de Janeiro, RJ, Brazil Setor de Mastozoologia, Museu Nacional/UFRJ Rio de Janeiro Brazil

**Keywords:** Cytochrome *b*, genetic diversity, morphology, phylogeography

## Abstract

*Bassaricyon* (Carnivora, Procyonidae) is a little-known group of small Neotropical carnivores from central and northern South America. Despite their charismatic nature, they are difficult to spot in the field, and they are confused with another procyonid, the kinkajou. This poorly studied genus comprises four species: *B. gabbii*, *B. alleni*, *B. medius*, and *B. neblina*, the latter with two subspecies. In this study, the genetic diversity of *Bassaricyon* from eastern Amazonia was explored based on the mitochondrial gene cytochrome *b*. A maximum-likelihood analysis confirms the taxa recognized at the species and subspecies levels and shows that populations from the Guiana Shield (GS) are well structured relative to the lineage from western Amazonia (WA), with genetic distances among them comparable to those of other *Bassaricyon* subspecies. A median-joining network showed five median vectors between *B. alleni* lineages, more than between *B. medius* and *B. neblina* subspecies (*n* = 1); additionally, there are at least 10 nucleotide substitutions between them, suggesting a considerable difference between these lineages. In the last review of the genus, the estimated divergence time between *B. alleni* from western Amazonia and Guiana Shield was slightly less than that between *B. neblina* and *B. medius* subspecies, suggesting less time to differentiate. We collected *Potos flavus* in the two municipalities sampled; however, in different localities, suggesting different habitat specificities. These results, together with morphological differences among *B. alleni* lineages, suggest a subspecies taxonomic status for the Guiana Shield population, *B. alleni beddardi*.

## Introduction

*Bassaricyon* J.A. Allen, 1876 (Carnivora, Procyonidae) is found in the forests of Central America and northern South America. Despite the charismatic nature of species in this genus, they are a little-known group of small Neotropical carnivores, not easy to see in the field, and confused with the kinkajou. A revision of the genus (Helgen et al. 2013) recognized four species: *Bassaricyon gabbii* J.A. Allen, 1876, *Bassaricyon alleni* Thomas, 1880, *Bassaricyon medius* Thomas, 1909, and *Bassaricyon neblina* Helgen et al., 2013 with *Bassaricyon beddardi* Pocock, 1921 as a junior synonym of *B. alleni*. *Bassaricyon* diversification is relatively recent, around 10.2 Ma. The earliest divergence within the genus (*B. neblina* and other *Bassaricyon*) occurred 3.5 Ma (CI = 2.1–5.2 Ma), whereas the separation between subspecies occurred more recently (around 0.25 Ma) (Koepfli et al. 2007; Helgen et al. 2013).

In the present study, genetic diversity analyses of *B. alleni* were conducted using the mitochondrial gene cytochrome *b*, including all species of the genus, and new specimens from the Brazilian Amazon, to investigate the taxonomic status of beddardi.

## Materials and methods

**Figure 1.**
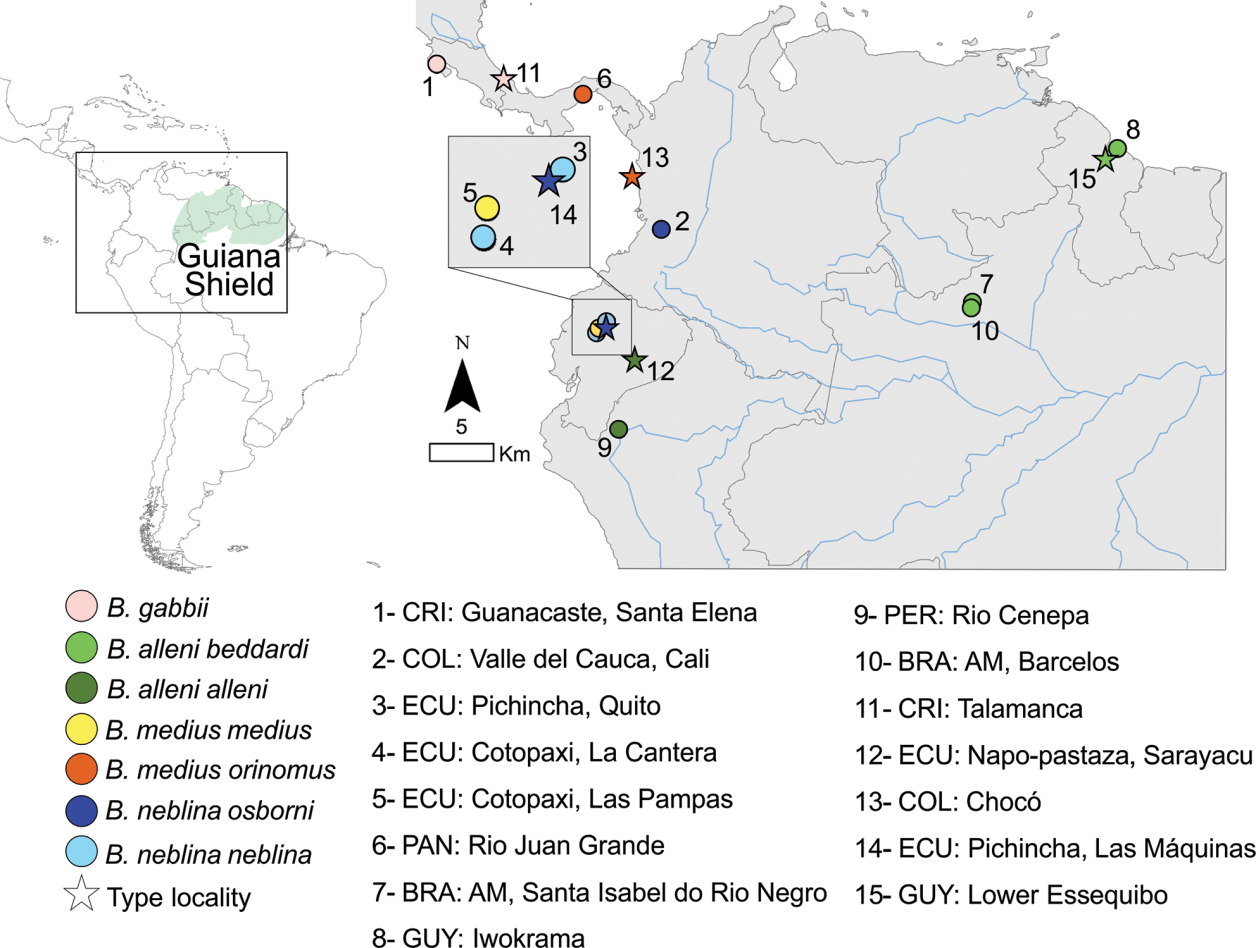
Map with sampling (circles) and type localities (stars) of *Bassaricyon*. The South American map (left) shows the Guiana Shield (adapted from de Alkmim 2015).

Four specimens were obtained in the Brazilian Amazon in 1998 and 2001 (Fig. 1). These specimens were collected and deposited in the mammal collections of Museu Nacional (**MN**), Universidade Federal do Rio de Janeiro, Brazil, including: municipalities of Barcelos on the left bank of the Rio Padauari, Igarapé Japomeri (males MN69145/field number CRB2210/GenBank PZ530650 and MN69150/ CRB2216/ PZ530649, and female MN69149/ CRB2215/ PZ530651), 00°20'51"N, 64°00'28"W, and Santa Isabel do Rio Negro on the right bank of the Rio Padauari, Piaçabal Ucuqui (male MN50186/ CRB1488/ KX756273), 00°18'10.4"N, 64°01'41.8"W in the Brazilian state of Amazonas; these two localities are far from one another and likely represent unrelated specimens.

**Figure 2.**
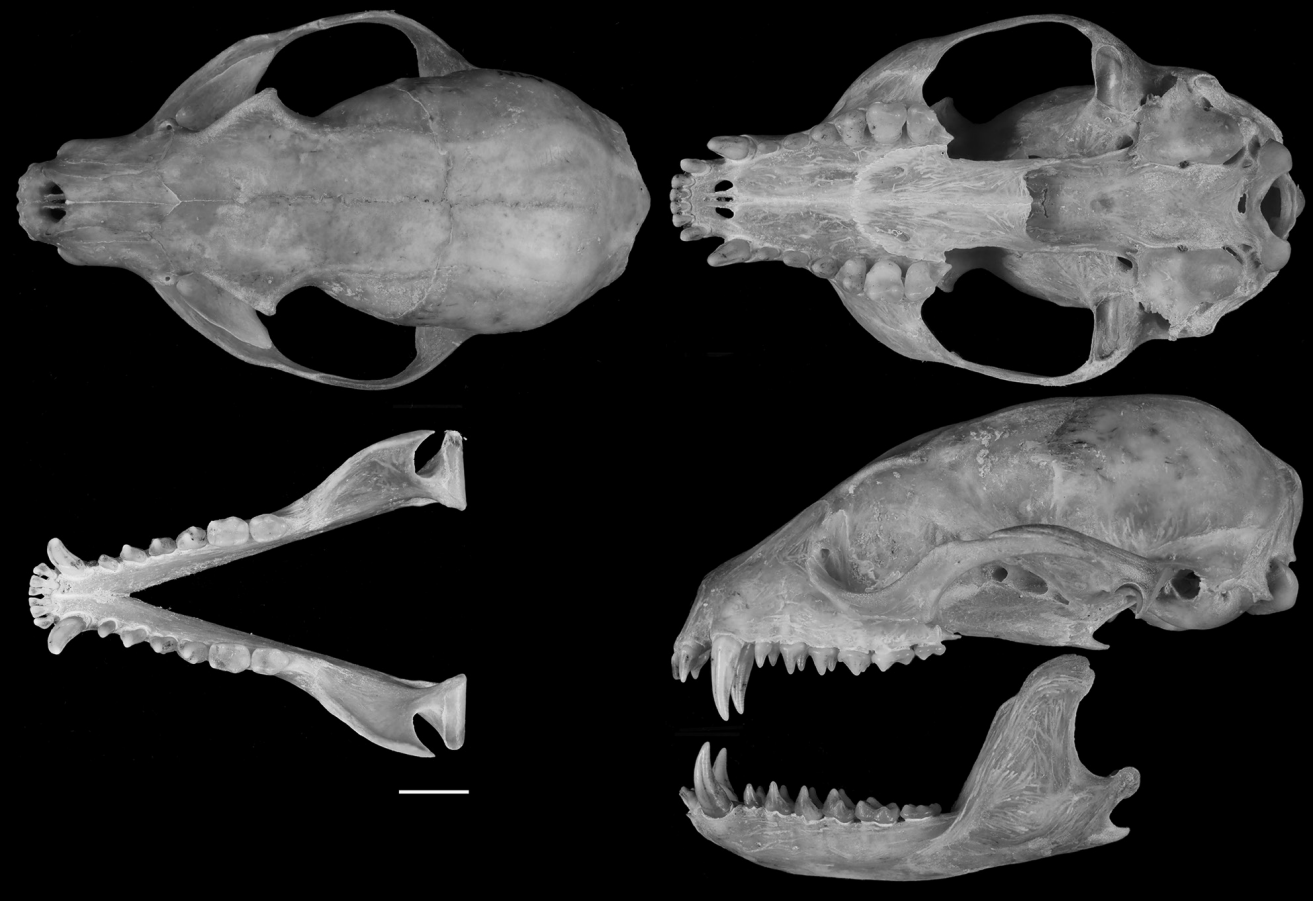
Skull of *Bassaricyon* from the Guiana Shield (MN 69150). Scale bar: 1 cm.

We compared the collected specimens (Fig. 2) with *B. alleni* and *B. beddardi* described and depicted by Helgen et al. (2013) and Pocock (1921). The cytochrome *b* gene was amplified with primers Mod-L14724 (Ferracioli et al. 2023) and CitbRev (Anderson and Yates 2000) and sequenced with the same primers and Citb-Sotin1 and Citb-Sotin2 (Cassens et al. 2000) under the following conditions: initial denaturation at 94 °C for 2 min, followed by 35 cycles at 94 °C for 30 s, 54 °C for 30 s, 72 °C for 90 s, and a final extension at 72 °C for 5 min. Sequences were aligned using Mega X (Kumar et al. 2018) with nine other sequences from GenBank: *B. alleni* (DQ660299, EF107710), *B. medius* (EF107706, EF107707, MK144297), *B. neblina* (EF107708-9) and *B. gabbii* (JX948744, DQ660300, EF107703-4, X94931). *Bassaricus* (AF498159, DQ660301) and *Potos* (EF107705) were included as outgroups. A maximum-likelihood (ML) tree and a median-joining (MJ) network were obtained using IQ-Tree v. 1.6.7 (Minh et al. 2013) and NETWORK (Bandelt et al. 1999), respectively.

## Results

*Bassaricyon alleni* specimens were collected by resident people close to “piaçabais”, between the floodplain and dryland forest. The skull (Fig. 2) of the specimen from the Guiana Shield (GS) showed qualitative differences in relation to the skull of *B. alleni* from western Amazonia (WA) in the following characters: (a) nasal short, exposing the entire incisive foramen in dorsal view, whereas in *B. gabbii* the caudal end of the incisive foramen cannot be seen in this view; (b) distance between the pterygoid process and the postglenoid process smaller than in *B. alleni*, but similar to *B. gabbii*, (c) supra-occipital process less developed than in *B. alleni* but similar to *B. gabbii*, (d) projection of the palate after the 3^rd^ molar parallel along its entire length, while it is convergent posteriorly in *B. alleni*, and (e) frontal spine broad at the base, while it is considerably more tapered in *B. alleni*. Furthermore, the shape of the mesopterygoid fossa is variable; it is U-shaped in old specimens and W-shaped in younger specimens. During these expeditions, resident people also collected the same number of *Potos flavus* in the municipalities of Barcelos (*n* = 3, along the Aracá River and along the road from Barcelos to Caurés River: GenBank KX756245, KX756247, KX756248) and Santa Isabel do Rio Negro (*n* = 1, Preto River: GenBank KX756246).

**Figure 3.**
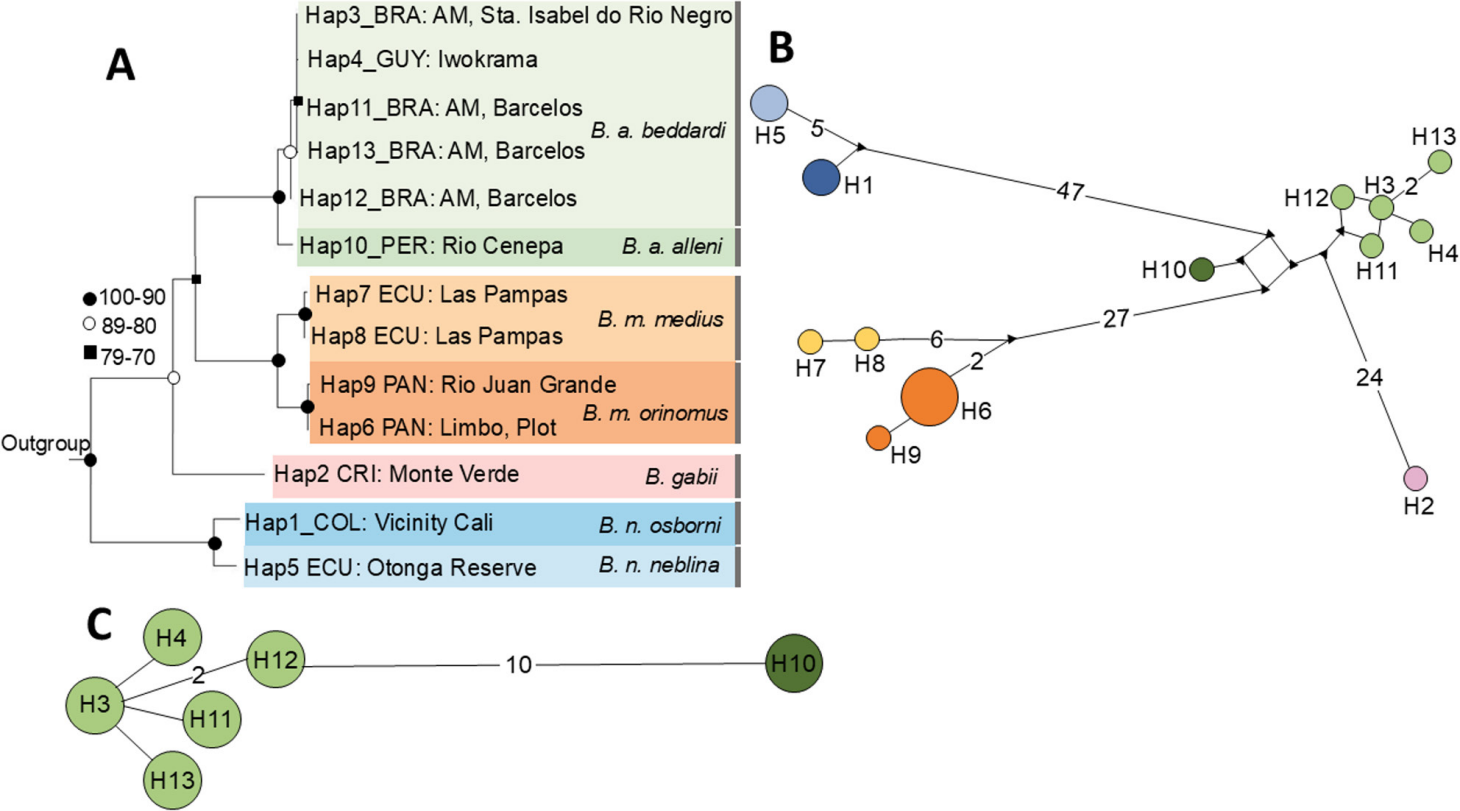
Relationships among *Bassaricyon* species. **A**. Maximum-likelihood tree with cytochrome *b*; symbols near nodes are bootstrap values; **B**. Median-joining network with *B. alleni* (green), *B. gabbii* (pink), *B. medius* (orange), *B. neblina* (blue); **C**. Median-joining network of *B. alleni*. Circles are haplotypes, with size proportional to sequences shared; triangles are median vectors; numbers near lines are nucleotide substitutions higher than one.

The ML tree showed the genus divided into four clades (Fig. 3A), corresponding to *B. gabbii*, *B. alleni*, *B. medius*, and *B. neblina*. The last three species are further each divided into two lineages. Kimura 2-parameter genetic distance between *Bassaricyon* species ranged from 5.5 to 9.7% (Table 1). MJ recovered the topology of ML and showed that the WA and GS lineages are separated by 10 nucleotide substitutions (Fig. 3C).

**Table 1.** Kimura 2-parameter (%) genetic distances within and among *Bassaricyon* species and subspecies, with GenBank or museum number, taxon, and country of collection. Colour cells represent each lineage, and bold numbers represent the distance between subspecies.

	**1**	**2**	**3**	**4**	**5**	**6**	**7**	**8**	**9**	**10**	**11**	**12**	**13**	**14**	**15**
1. MN69145*B. a. beddardi* Brazil															
2. MN69150*B. a. beddardi* Brazil	0.3														
3. KX756273*B. a. beddardi* Brazil	0.2	0.1													
4. MN69149*B. a. beddardi* Brazil	0.4	0.3	0.2												
5. EF107710*B. a. beddardi* Guyana	0.4	0.2	0.2	0.4											
6. DQ660299*B. a. alleni* Peru	**0.9**	**1.3**	**1.1**	**1.3**	**1.3**										
7. DQ660300*B. orinomus* Panama	5.8	5.9	5.9	6.1	6.0	5.5									
8. EF107703*B. orinomus* Panama	5.9	6.2	6.1	6.4	6.3	5.7	0.3								
9. EF107704*B. orinomus* Panama	6.1	6.3	6.2	6.4	6.3	5.8	0.4	0.2							
10. MK144297*B. orinomus* Panama	5.8	5.9	5.9	6.1	6.0	5.5	0.0	0.3	0.4						
11. EF107706*B. medius* Ecuador	6.3	6.6	6.4	6.6	6.5	5.8	**1.5**	**1.8**	**1.9**	**1.5**					
12. EF107707*B. medius* Ecuador	6.4	6.7	6.5	6.7	6.6	5.9	**1.6**	**1.9**	**2.0**	**1.6**	**0.1**				
13. EF107708*B. neblina* Ecuador	9.1	8.8	9.3	9.5	9.7	9.5	8.5	8.8	8.8	8.5	9.1	9.2			
14. EF107709*B. neblina* Ecuador	9.1	8.8	9.3	9.5	9.7	9.5	8.5	8.8	8.8	8.5	9.1	9.2	0.0		
15. X94931*B. osborni* Colombia	8.9	8.2	9.2	9.4	9.6	9.2	8.4	8.7	8.7	8.4	8.9	**9.1**	**1.5**	**1.5**	
16. JX948744*B. gabbii* Costa Rica	5.9	4.3	5.8	6.1	5.7	5.9	6.3	6.6	6.4	6.3	6.2	6.1	8.6	8.6	9.0

## Discussion

Our analyses based on morphological and molecular data show that the diversity within *Bassaricyon* is greater than previously thought, suggesting the taxonomic status of subspecies for GS population (*B. alleni beddardi*), and suggest that *Bassaricyon* and *Potos* occur in different habitats. The four specimens of *Potos flavus* from Barcelos and Santa Isabel do Rio Negro (Padauari and road from Barcelos to Caurés River; Nascimento et al. 2016) were never found at the same localities of *Bassaricyon* (Aracá and Preto rivers), which suggests differing habitat preferences.

The differences in pelage and skull characters identified between *B. alleni* populations from WA and GS confirm those differences in pelage colouration between *B. alleni* and *B. beddardi* already postulated by Pocock (1921); i.e., the pelage is darker in the GS/beddardi lineage, and the skull is a little narrower and with a more depressed muzzle in GS/beddardi specimens.

Our study confirms previous taxonomic arrangements (Fig. 3; Nyakatura and Bininda-Emonds 2012; Helgen et al. 2013) and suggests that *B. alleni* should be divided into two populations, GS and WA (Fig. 3). Furthermore, the GS population and the WA population are well structured, and the genetic distances observed between these lineages (0.9–1.3%) are comparable to the genetic distances observed for other *Bassaricyon* subspecies, which ranges are 1.5–2% between *B. medius medius* and *B. m. orinomus*, and 1.5% between *B. n. neblina* and *B. n. osborni*. In the last review of the genus, samples from both lineages of *B. alleni* were used, and the time of divergence between them was slightly lower than that between *B. neblina* and *B. medius* subspecies (Helgen et al. 2013), showing that these lineages had less time to differentiate. Despite this, the number of median vectors between *B. alleni* lineages (*n* = 5) is higher than that between *B. medius* and *B. neblina* subspecies (*n* = 1), suggesting a considerable difference between these lineages. These median vectors represent inferred ancestral haplotypes that connect sampled lineages, likely present in the population’s past but possibly lost or not sampled in the present population.

The type locality of *B. alleni* is Sarayacu, Upper Pastasa River, Ecuador, and that of *B. beddardi* is Bartica Woods, Essequibo River, Guyana (Helgen et al. 2013). The characters of Guiana Shield specimens are similar to those of the holotype of *B. a. beddardi* (figured and discussed by Pocock 1921), and, together with genetic differences, support the taxonomic status of *B. a. beddardi* as a subspecies. It is interesting that of the 294 species of mammals reported from the Guianas, most (94%) are found in the lowlands. Guiana endemic species account for 14% of mammalian biodiversity (Lew and Lim 2019); however, this publication did not consider *Bassaricyon* or *Potos flavus* to occur in the Guiana Shield.

This *Bassaricyon* population from the GS has already been considered as a species (Sampaio et al. 2011), and part of its distribution overlaps with the deforestation arc in southwestern Brazil. As such, this indicates a threat, particularly as the species is strictly arboreal (Sampaio 2013). A study in the Brazilian Amazon showed evidence suggesting that populations of the “gogó-de-sola” (identified as *B. beddardi*) declined from 1994 to 2009, while its direct competitors, *Aotus trivirgatus* (night monkey) and *Potos flavus* (kinkajou), appear to have undergone the opposite process (Pontes and Beisiegel 2013). Therefore, with the demonstration that the *Bassaricyon* from the GS deserves subspecies status, more attention should be given to its conservation status. This is especially true given that fewer specimens of the Guiana population are available in Brazilian collections, suggesting a low-density population, restricted habitat use, or rarity.

## Additional information

### Artificial Intelligence (AI) use

The authors accept full responsibility for the content of the manuscript, including the disclosure of any use of AI.

No AI tools were used in the preparation of this manuscript.

### Author ORCIDs

C.R. Bonvicino https://orcid.org/0000-0002-1948-7643

M.J. Cardoso https://orcid.org/0009-0006-5865-5968

F. Souza-Gudinho https://orcid.org/0000-0002-2510-0337

### Data availability

All of the data that support the findings of this study are available in the main text.
